# Biomarker Identification through Multiomics Data Analysis of Prostate Cancer Prognostication Using a Deep Learning Model and Similarity Network Fusion

**DOI:** 10.3390/cancers13112528

**Published:** 2021-05-21

**Authors:** Tzu-Hao Wang, Cheng-Yang Lee, Tzong-Yi Lee, Hsien-Da Huang, Justin Bo-Kai Hsu, Tzu-Hao Chang

**Affiliations:** 1 Graduate Institute of Biomedical Informatics, College of Medical Science and Technology, Taipei Medical University, Taipei 110, Taiwan; b101105130@tmu.edu.tw (T.-H.W.); nathanlee@tmu.edu.tw (C.-Y.L.); 2 School of Medicine, College of Medicine, Taipei Medical University, Taipei 110, Taiwan; 3 Office of Information Technology, Taipei Medical University, Taipei 110, Taiwan; 4 Warshel Institute for Computational Biology, The Chinese University of Hong Kong, Shenzhen 518172, China; francis@saturn.yzu.edu.tw (T.-Y.L.); huanghsienda@cuhk.edu.cn (H.-D.H.); 5 School of Life and Health Science, The Chinese University of Hong Kong, Shenzhen 518172, China; 6 Department of Medical Research, Taipei Medical University Hospital, Taipei 110, Taiwan; 7 Translational Imaging Research Center, Taipei Medical University Hospital, Taipei 110, Taiwan; 8 Clinical Big Data Research Center, Taipei Medical University Hospital, Taipei 110, Taiwan

**Keywords:** prostate cancer, multiomics, autoencoder, deep learning, similarity network fusion, machine learning, prognosis prediction, recurrence prediction

## Abstract

**Simple Summary:**

Around 30% of men treated with adjuvant therapy experience recurrences of prostate cancer (PC). Current monitoring of the relapse of PC requires regular postoperative prostate-specific antigen (PSA) value follow-up. Our study aims to identify potential multiomics biomarkers using modern computational analytic methods, deep learning (DL), similarity network fusion (SNF), and the Cancer Genome Atlas (TCGA) prostate adenocarcinoma (PRAD) dataset. Six significantly intersected omics biomarkers from the two models, *TELO2*, *ZMYND19*, *miR-143*, *miR-378a*, cg00687383 (*MED4*), and cg02318866 (*JMJD6*; *METTL23*) were collected for multiomics panel construction. The difference between the Kaplan–Meier curves of high and low recurrence-risk groups generated from the multiomics panels and clinical information achieve *p*-value = 2.97 × 10^−15^ and C-index = 0.713, and the prediction performance of five-year recurrence achieves AUC = 0.789. The results show that the multiomics panel provided valuable biomarkers for the early detection of high-risk recurrent patients, and integrating multiomics data gave us the power to detect the complex mechanisms of cancer among the interactions of different genetic and epigenetic factors.

**Abstract:**

This study is to identify potential multiomics biomarkers for the early detection of the prognostic recurrence of PC patients. A total of 494 prostate adenocarcinoma (PRAD) patients (60-recurrent included) from the Cancer Genome Atlas (TCGA) portal were analyzed using the autoencoder model and similarity network fusion. Then, multiomics panels were constructed according to the intersected omics biomarkers identified from the two models. Six intersected omics biomarkers, *TELO2, ZMYND19, miR-143, miR-378a*, cg00687383 (*MED4*), and cg02318866 (*JMJD6*; *METTL23*), were collected for multiomics panel construction. The difference between the Kaplan–Meier curves of high and low recurrence-risk groups generated from the multiomics panel achieved *p*-value = 5.33 × 10^−9^, which is better than the former study (*p*-value = 5 × 10^−7^). Additionally, when evaluating the selected multiomics biomarkers with clinical information (Gleason score, age, and cancer stage), a high-performance prediction model was generated with C-index = 0.713, *p*-value = 2.97 × 10^−15^, and AUC = 0.789. The risk score generated from the selected multiomics biomarkers worked as an effective indicator for the prediction of PRAD recurrence. This study helps us to understand the etiology and pathways of PRAD and further benefits both patients and physicians with potential prognostic biomarkers when making clinical decisions after surgical treatment.

## 1. Introduction

Prostate cancer (PC) is the second most frequent cancer diagnosis made in men and the fifth leading cause of death worldwide, with a rapidly rising number of patients in the past few decades. Based on GLOBOCAN 2018 estimates [1], 1,276,106 new cases of PC were reported globally in 2018, with higher prevalence in developed countries. According to an estimate by the American Cancer Society [2], there were about 191,930 new cases of PC and about 33,330 deaths from PC in 2020 in the U.S.

At present, standard treatments of PC include a prostatectomy, radiation therapy, or both. Despite these aggressive approaches, 25–40% of treated men experience recurrences of PC [3]. R factors associated with PC recurrence include the prostate-specific antigen (PSA) level in serum, the Gleason score of the prostate specimen, the patient’s age, and the cancer stage. The most common early sign of recurrent PC is a rising serum PSA level. Although this requires regular postoperative follow-up, routine monitoring of the serum PSA is the mainstream method for detecting early PC recurrence in current clinical practice. As the data collecting technology advances with time, more and more omics data are being collected for cancer research, such as the Cancer Genome Atlas (TCGA), International Cancer Genome Consortium (ICGC), and Gene Expression Omnibus (GEO) databases [4,5,6]. With the help of this big data support, we can obtain better predictive performance results. Meanwhile, machine learning has been growing at an incredible pace these days, and many types of research using techniques of deep learning have been skyrocketing.

After a survey of previous works (shown in Table 1), we discovered that few studies analyzed the recurrence of PC with multiomics data using computational methods. Our study can fill in the gap of lacking prostate adenocarcinoma (PRAD)-related research using multiomics data.

Yousefi et al. [7] focused on using deep learning (DL) and Bayesian optimization methods for predicting cancer outcomes. By obtaining clinical and molecular data from multiple cancer datasets, including pan-glioma (LGG/GBM), breast (BRCA), and pan-kidney (KIPAN), from the Cancer Genome Atlas (TCGA), this study illustrated the success of transferring clinical information across diseases by deep survival models to improve prognostication. Chaudhary et al. [8] attempted to identify robust survival subgroups of hepatocellular carcinoma (HCC). Two optimal subgroups of patients were identified with significant survival differences, of which frequent *TP53* inactivation mutations, higher expression of stemness markers (*KRT19* and *EPCAM*) and the tumor marker *BIRC5*, and the activation of Wnt and Akt signaling pathways are associated with the more aggressive subtype.

Zhang et al. [9] adopted a DL algorithm, autoencoder, to integrate multiomics data obtained from TCGA, and the K-means clustering algorithm was applied to classify two subtypes with significant survival differences. In the end, this study indicated that in the ultra-high-risk subtype, the occurrence of amplification of the *MYCN* gene is more frequent, in agreement with the overexpression of *MYC/MYCN* targets in this subtype. Zhang et al. [10] illustrated an integrative framework to recognize ovarian cancer-related genetic and epigenetic features and to evaluate the causal relationships among these features based on TCGA data. They discovered a set of features of 13 hub genes, including *ARID1A*, *C19orf53*, *CSKN2A1*, and *COL5A2*, and two genes associated with glycoprotein synthesis, *PSG11* and *GALNT10*, that were highly accurately predictive of the overall survival times of ovarian cancer patients. Wang et al. [11] performed an innovative approach called similarity network fusion (SNF) to create a comprehensive view of a given disease with multiomics data by network constructions of samples for each input data type. They then fused the networks into a single network that represented the full spectrum of the underlying data. They discovered better outcomes for survival predictions and subtype identification compared to other integrative analyses using only a single data type.

Herein, we propose modern computational analytical methods, the autoencoder model, as our adopted DL algorithm, and SNF to create a comprehensive view of the connections among methylation-related gene expressions, micro (mi)RNA, and gene expressions, and differentiate patients at a high risk of recurrence with the prediction model to better predict the prognosis of PC. The autoencoder model is an artificial neural network comprised of an encoder and a decoder. The most important attribute of the autoencoder model [12] is that it can be used to learn a compressed representation that better captures properties that reflect the variety of patients’ prognoses. On the other hand, SNF is a computational method for data integration, which iteratively integrates each individual network into a single fused network to create a comprehensive view of a biological process in a given disease.

## 2. Materials and Methods

**Figure 1:**
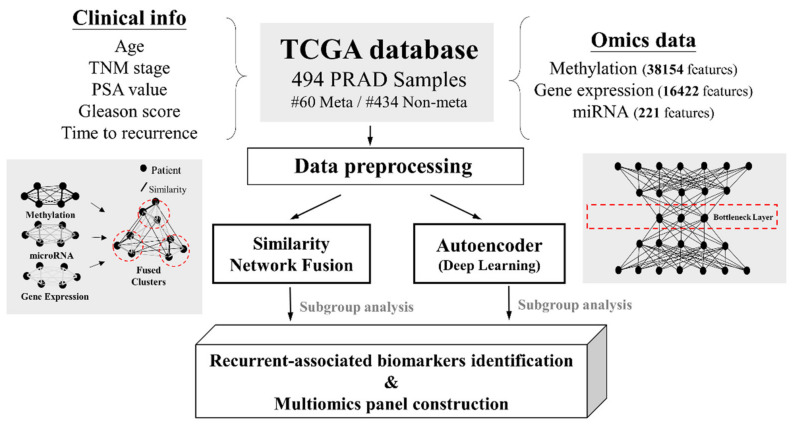
Overview of the study workflow. TNM, tumor, node, metastasis status; PSA, prostate-specific antigen.

We applied two innovative computational analyses, the autoencoder model and SNF, on PRAD patients to help better predict disease recurrence. Our workflow of methods (as shown in Figure 1) was divided into two parts: one part focused on identifying recurrent biomarkers based on their integration of omics information, and the other constructed a prediction model according to recurrence-associated omics features.

### 2.1. Data Collection and Preprocessing

The data we used were extracted from TCGA portal (https://tcga-data.nci.nih.gov/tcga/ (accessed on 15 November 2020)). The dataset was composed of 494 PRAD samples with all level-3 omics data and clinical information, including age, time to recurrence, TNM stage, PSA value, and Gleason score. (Table S1).

Omics features were omitted if zero or if values were missing. Each sample was normalized using the standardNormalization function in the SNFtool package [11] before further calculation. Integration of the input dataset was conducted with R programming software.

### 2.2. SNF Construction

First, SNF was used to construct sample similarity matrices for each of the omics data types (gene expression, methylation-related genes, and miRNA) using pairwise correlations. Then, we set the number of neighbors to 20 and the hyperparameter sigma to 0.5 to transform sample similarity matrices to sample similarity graphs where nodes were samples and edges represented samples’ pairwise similarities.

Next, we ran the sample network fusion for 20 iterations, updating each of the sample similarity networks with information from the other networks, making them more similar. At last, the final fused network of samples, to which the SNF process had converged, generated optimal numbers of subgroups among patients according to spectral clustering.

We implemented the SNFtool package in R programming software to conduct the graphical integration analysis [11].

### 2.3. DL Framework

Three preprocessed TCGA PRAD omics datasets with a total of 494 patients were stacked into a new matrix before being input into the autoencoder model. Next, we applied a classic autoencoder model with three hidden layers (of 500, 200, and 500 nodes, respectively), of which the 200-node bottleneck layer represented new features. Then, among these 200 new features, we selected 53 features (with the Cox-PH model, *p* < 0.05) that were associated with the attribute of time to recurrence.

We trained the autoencoder model using a gradient descent algorithm, the tanh activation function, five epochs, a batch size of 32, and a learning rate of 1 × 10^−6^. The parameters of L1 and L2 regularization were set to 0.0001 and 0.001, respectively.

The data integration analysis of the autoencoder model was implemented in R programming software with the ANN2 package [9].

### 2.4. Identification of Recurrence-Associated Variables and Subgroups

Univariate Cox regression analysis: after the autoencoder model reduced the initial number of features to 200 new nodes acquired from the bottleneck layer, we built a univariate Cox-PH model and selected nodes that were significantly associated with time to recurrence (*p* < 0.05). We built a Cox-PH model using the R survival package.

K-means clustering algorithms for the autoencoder model: we then used these recurrence-associated nodes to cluster samples using the K-means clustering algorithm. The optimal number of clusters was determined according to two metrics: the silhouette index [13] and elbow methods [9].

Spectral clustering algorithms for SNF: as for the SNF portion, we applied the spectral clustering function in the R SNFtool package to cluster the samples. The optimal number of clusters was estimated with two heuristics: Eigen-gaps and rotation cost methods.

### 2.5. Functional Enrichment Analysis of Recurrence-Associated Variables

Identification of differentially expressed omics data: to identify genes, methylation-related genes, and miRNAs that were differentially expressed between the high-risk and low-risk subgroups of recurrence, we calculated the average value for each feature in each subgroup. Next, we implemented the Wilcoxon rank-sum test in R programming to search for the top differentially expressed features between the two subgroups.

Functional analysis: GeneGO-MetaCore [14] (http://www.genego.com/metacore.php (accessed on 25 January 2021)): differentially expressed genes (DEGs) between the two subgroups were uploaded to MetaCore from Clarivate. The database in MetaCore is unique and highly accurate, which is manually corrected. *p* values were calculated by the hypergeometric distribution in MetaCore to assess the statistical significance of the enrichment pathways and diseases (by biomarkers) and multiple test corrections using false discovery rate adjustments.

### 2.6. Evaluation of the Discrimination Power between the Autoencoder Model and SNF

Two metrics (C-index and log-rank *p* values) were applied for the purpose of assessment. They could genuinely reflect the predictive accuracy of the recurrence in our identified subgroups.

Concordance index: the concordance index (C-index) is defined as the proportion of concordant pairs divided by the total number of possible evaluation pairs [15], and it is based on the Harrell C statistic [16]. The method for calculating the C-index is to randomly pair up samples from the data, and if one with an actual shorter survival time presents a shorter predicted survival time or lower predicted survival probability than the other, this means that the prediction result is in conformity with the actual result. We constructed the Cox-PH model and calculated the C-index using the R survival package. We assessed the predictive accuracy of the recurrence subgroup according to the C-index when higher values indicated better discrimination.

Log-rank p value of the Cox-PH regression: the log-rank test is a statistical test to compare the survival times between two or more groups. Kaplan–Meier survival curves were plotted based on the two risk groups, and the log-rank *p* values of differences in survival between the curves were calculated with the R survminer package [17].

### 2.7. Construction of the SVM Classifier Based on the Labeled Subgroup

This data partitioning aimed to evaluate the robustness of the SVM classifier. Labels of TCGA samples were generated from K means clustering using nodes of the autoencoder model built with all of the samples. Next, we selected the top omics features that were most correlated with the subgroup labels based on the Wilcoxon rank-sum test, and then combined these top-selected omics data together as one dataset named multiomics features. The default selection numbers were set to 100 for gene expression, 100 for methylation-related genes, and 50 for miRNAs. Next, we built supervised classification classifiers using the SVM algorithm with different combinations of multiomics data and clinical data, including the Gleason score, age, and TNM (tumor, node, metastasis status) stage.

We used a 5-fold cross-validation method to partition TCGA dataset as follows: we first randomly split the 494 samples from TCGA into five folds. One of the five folds was used as the test set and the remaining four folds as the training set. For each training set, an SVM classifier was built to predict the labels of the test set. This data partitioning aimed to assess the robustness of the SVM classifiers. Therefore, we performed 10 repetitions and obtained the mean value representing the average prediction accuracy.

### 2.8. Multiomics Panel Construction for PRAD Recurrence Prediction

Univariate Cox regression was performed on the intersected omics data between the DL and SNF models, of which those found significantly associated with recurrence were retained for further analysis. Next, we applied multivariate Cox regression on the selected omics data to construct a linear risk-score model. The risk score for each sample was calculated using the following formula; where βi indicates the coefficients evaluated with omics expression and xi refers to the relative omics expression level.

Risk score = ∑inβi*xi

Finally, the samples were divided into low- and high-risk groups according to the cutoff risk score calculated by R survMisc package.

## 3. Results

### 3.1. Outcome of the SNF Analysis

#### 3.1.1. Two Differential Recurrence-Risk Subgroups were Identified in TCGA Three-Omics Data

**Figure 2:**
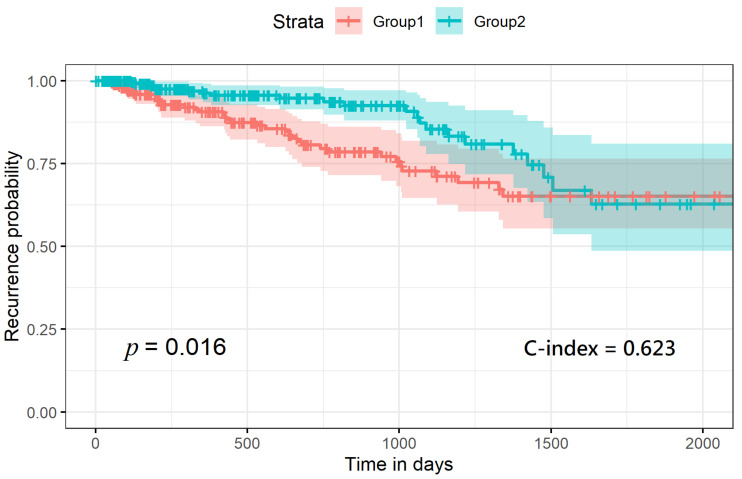
Recurrence-risk curves according to the labels generated from the similarity network fusion.

The optimal estimated number of clusters from the SNF analysis was two, according to the calculation of both the Eigen-gap algorithm [18] and the rotation-cost algorithm [19] for the given materials of the 494 PRAD patient samples. As shown in Figure 2, with each labeled subgroup, we combined samples with their time-to-recurrence value and ran the ggplot2 package in R programming to plot out the recurrence-risk curves. As a result, we obtained two curves with a *p* value of 0.016 and C-index of 0.623.

#### 3.1.2. Differential Expression Analysis of Each Omics Dataset

We sorted out the top 100 DEGs and methylation-related genes, as well as the top 50 differentially expressed miRNAs between the subgroups. A detailed list of the top differential genes is shown in Table S2.

### 3.2. Outcome of the Autoencoder Model Analysis

#### 3.2.1. Performance of the Autoencoder Model with Different Hyper-Parameters

**Figure 3:**
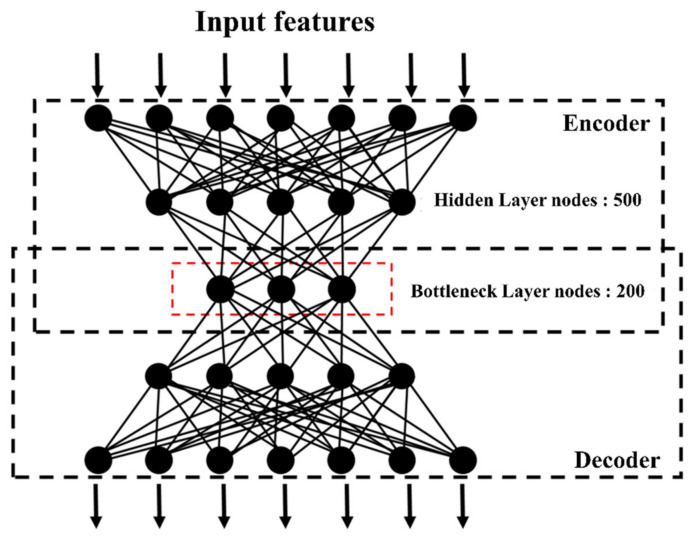
Architecture of the autoencoder model we adopted in this study.

To obtain the best performance from the training algorithm, we fit the autoencoder model with different hyper-parameters. Figure 3 shows the architecture of the autoencoder model we used. There were three parameters targeted: the number of bottleneck nodes, the number of epochs, and the number of hidden layers. Then, we compared the different-parameter-generated autoencoder models in terms of prognostic performance measured by the C-index. As shown in Table 2, more hidden layers or a higher number of bottleneck layer nodes usually decreased the performance instead of improving it. Oddly, when it came to the number of epochs, the performance did not accordingly increase or decrease in a trend. At last, we selected the one with the highest C-index (of 0.684) as our autoencoder model, which was constructed of three hidden layers, and 500, 200, and 500 nodes, respectively, with the parameter of epochs set to five.

#### 3.2.2. Two Recurrence-Risk Subgroups Identified

**Figure 4:**
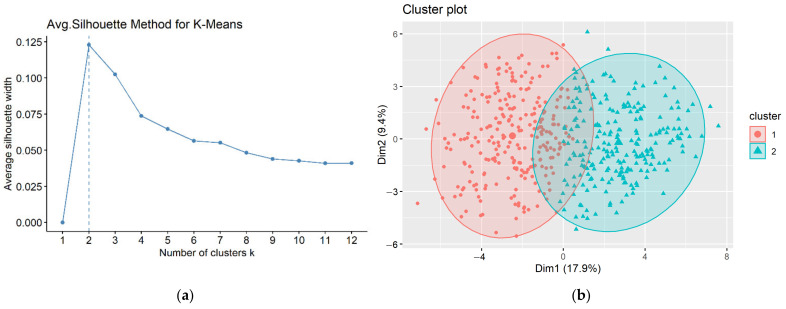
(**a**) The silhouette index plot indicating that the optimal number of clusters was two, (**b**) clustering plot of patients according to the K-means algorithm.

We built a univariate Cox-PH regression on each of the 200 nodes extracted from the bottleneck layer to identify those which were significantly (*p* < 0.05) associated with time-to-recurrence. Eventually, 53 nodes were shown to be significant. These 53 nodes were subsequently utilized to cluster samples using the K-means algorithm. To determine the optimal number of clusters, we performed the silhouette index, as shown in Figure 4a. We found that K = 2 was the best number of clusters with the highest score. Therefore, TCGA PRAD samples were dichotomized.

**Figure 5:**
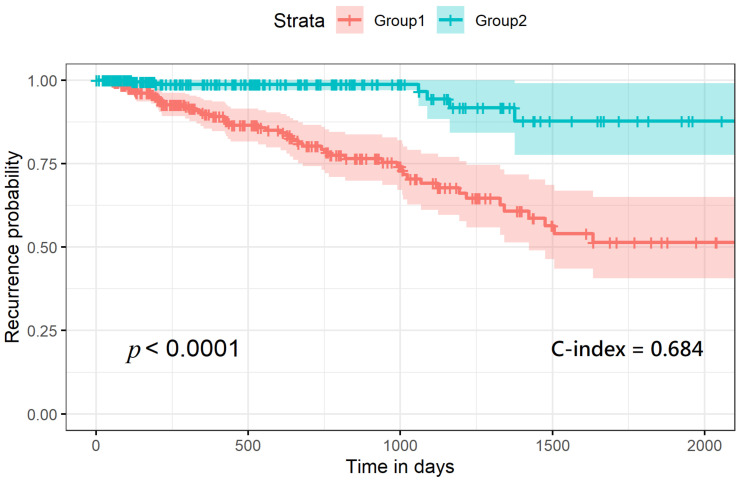
Recurrence-risk curves according to the labels derived from the autoencoder model.

Furthermore, we assessed prognostic differences between these two subgroups with a recurrence-risk analysis, and the difference between the two subgroups was extremely significant (log-rank *p* = 7 × 10^−8^), with a C-index of 0.684 (Figure 5).

#### 3.2.3. Differential Expression Analysis between the Two Subgroups

After obtaining labels generated from K-means clustering, we listed the top 100 DEGs and methylation-related genes, as well as the top 50 differentially expressed miRNAs between the subgroups, as shown in Table S3.

### 3.3. Validation of the Robustness of the SVM Classifier

In order to construct the recurrent prediction model using the SVM algorithm, we adopted subgroups from the autoencoder model for labeling the risk of samples due to its highly distinguishing performance (C-index) compared to the SNF.

To test the robustness of the classification on predicting prognoses, we validated the SVM classifiers on fivefold cross-validation with 10 repetitions on the testing dataset. As shown in Table 3, we first trained the SVM classifier with the 53 autoencoder model-generated nodes. The prediction accuracy on the testing dataset was 97.1%, with 97.0% sensitivity and 97.2% specificity. Next, we input the multiomics data composed of the top 100, 100, and 50 DEGs from the expression, methylation, and miRNA datasets, respectively, including 250 features in total, into the SVM classifier. The prediction accuracy was 90.8%, with 93.8% sensitivity and 87.4% specificity.

Moreover, we further assessed the performance of the prediction accuracy by adding additional clinical variables as features to build other SVM classifiers (Table 3). As a result, we discovered that adding clinical variables, including the Gleason score, age, and TNM stage, helped to improve the prognostic prediction accuracy. The combination of a multiomics dataset (250 features) with all clinical information altogether produced 93.7% accuracy, with 95.6% sensitivity and 91.5% specificity, on the testing dataset. Moreover, we also evaluated the predicting performance of individual clinical information combined with the multiomics dataset. The accuracy of the SVM classifier increased to 94.3%, with 96.5% sensitivity and 91.7% specificity, when the stage was input with the multiomics dataset, which generated the highest prediction accuracy among all clinical features. Adding the SVM classifiers of age or Gleason score also showed good prediction results, with 90.7% accuracy, 93.6% sensitivity, and 87.4% specificity and 90.6% accuracy, 93.2% sensitivity, and 87.6% specificity, respectively.

Overall, the SVM classifier using the autoencoder model-generated nodes had the best prediction accuracy, and outperformed the ones using multiomics features, with or without adding clinical features.

### 3.4. Intersected DEGs between the Autoencoder Model and SNF

After acquiring results from each novel computational analysis, we further conducted a comprehensive comparison of the top 100 DEGs, 100 methylation-related genes, and the top 50 selected miRNAs between the two models.

The results showed that there are 21 genes (*LSM7, PAXX, PPP1R35, MHENCR, PSMG3, ATP5MPL, POLR2H, TELO2, PFDN6, PLEKHJ1, STX10, ZMYND19, FYCO1, PARVA, NFE2L2, MBNL2, LPP, ELF1, RNF185, IL6ST, PARM1*), 3 methylation-related genes (cg00687383 (*MED4*), cg02318866 (*JMJD6; METTL23*), cg02978959 (CTC-444N24.6; ZNF460) and 33 miRNAs (*hsa-mir-143, hsa-mir-379, hsa-mir-1247, hsa-mir-452, hsa-mir-133a-2, hsa-mir-133a-1, hsa-mir-1-1, hsa-mir-1-2, hsa-mir-221, hsa-mir-152, hsa-mir-328, hsa-mir-505, hsa-mir-324, hsa-mir-107, hsa-mir-136, hsa-mir-181b-2, hsa-mir-128-2, hsa-mir-181b-1, hsa-mir-193b, hsa-mir-381, hsa-mir-222, hsa-mir-139, hsa-mir-455, hsa-mir-132, hsa-mir-134, hsa-mir-127, hsa-mir-365b, hsa-mir-365a, hsa-mir-574, hsa-mir-374b, hsa-mir-148b, hsa-mir-193a, hsa-mir-378a*) found intersected between the two models. Therefore, we extracted these omics features from each model and compared their gene expression profiles within the subgroups. Finally, we discovered that each gene expressed the identical regulatory direction in both models and further reinforced that these intersected omics data could be potential biomarkers for PRAD recurrence prediction. The comparison of the gene expression profiles between the two models is shown in Table S4.

### 3.5. Prognostic Multiomics Panel Construction

**Figure 6:**
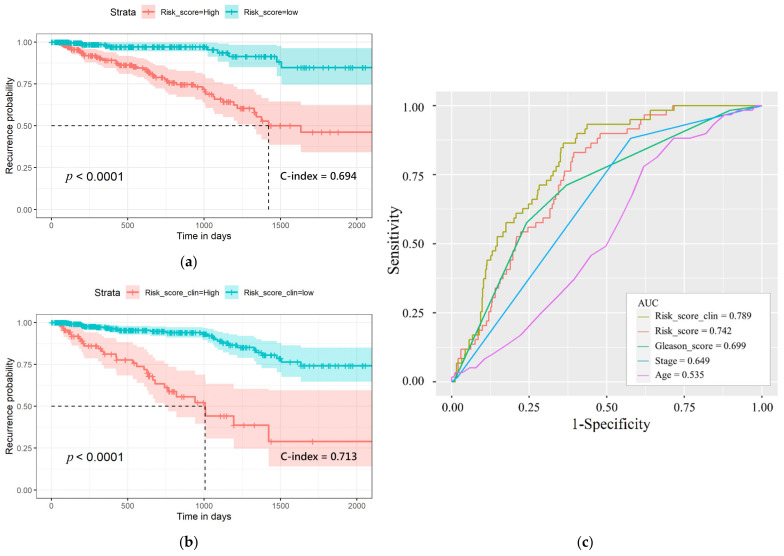
(**a**) Recurrence-risk curves according to high and low risk score. (**b**) Recurrence-risk curves according to risk score combined with clinical information. (**c**) The 5-year survival receiver operating curve of risk score and other clinical data and their AUC.

*TELO2, ZMYND19, miR-143, miR-378a,* cg00687383 (*MED4*), and cg02318866 (*JMJD6*; *METTL23*), and the first and second most significant *p*-value from each intersected omics data of the two models were identified for multiomics panel development. According to the results shown in Figure 6, the recurrence in the high-risk-score group was significantly shorter compared with the low-risk score group (*p*-value = 5.33 × 10^−9^, C-index = 0.694). The performance of discrimination power of our multiomics panel was better than the previous study using gene expression data only (*p*-value = 5 × 10^−7^) [20]. Additionally, the multiomics panel combined with clinical information also generated good performance in differentiating recurrence-risk subgroups (C-index = 0.713, *p*-value = 2.97 × 10^−15^). Then, the 5-year receiver operating characteristic (ROC) curve was graphed to compare the prognostic value of the multiomics panel and the multiomics panel adding clinical data and other clinical information. The area under the curve of the ROCs (AUC) of age, Gleason score, cancer stage, the multiomics panel, and the multiomics panel with clinical data were 0.535, 0.699, 0.648, 0.742, and 0.789, respectively. These results indicate that the risk score is better at predicting recurrence-risk than the other clinical information. Additionally, the omics features selected according to the intersected data of the two models are robust for PRAD recurrence prediction.

## 4. Discussion

### 4.1. Potential Recurrence Biomarkers of PRAD from the Autoencoder Model and SNF

Among the intersected omics features, *TELO2* and *ZYMND19* are the most significant DEGs. Guo et al. [21] revealed that *TELO2* was significantly upregulated in colorectal cancer (CRC), which was concordant with the regulatory pattern in our study. Additionally, the significant restraints of the growth, cell cycle, and metastasis of CRC cells manifest after *TELO* inhibition, indicating that *TELO2* promotes tumor progression. Iddawela et al. [22] used the top nine genes (including gene *ZMYND19*) correlated with *H2AFX* expression to generate a 10-gene signature. Given that patients with poor outcome can be defined by their DNA damage signature, these gene signatures can become potential prognostic markers for early PC patients’ treatment decisions.

In addition, the conclusions of previously related studies were also concordant with our results for genes, which were correlated with the regulation of PC. Fan et al. [23] indicated that *POLR2H* played a key role regarding the occurrence of PC. The result also showed that *POLR2H* was significantly upregulated in PC, which implied that this gene might be a potential biomarker for prognosis, diagnosis, and drug targets. Bii et al. [24] identified *MBNL2* as a candidate PC progression gene, which was downregulated to mediate the progression of androgen-independent PC. Hua et al. [25] also showed that *MBNL2* was downregulated in PC samples compared to normal samples, and the confirmation of the prognostic value was comprehensively evaluated by correlations with pathological T staging, the pathological grade, and Gleason score, revealing a good diagnostic and prognostic value for PC.

Budka et al. [26] suggested that *ELF1* was the most generally downregulated ETS factor in primary prostate tumors, and in the case of metastatic disease, the expression of *ELF1* also decreased. *ELF1* was negatively correlated with PC progression. Fladeby et al. [27] demonstrated that *PARM-1* is a novel androgen-regulated gene, highly expressed in androgen-dependent cancer xenografts. They further revealed the phenomenon of the increasing growth of PC cells when forcibly elevating the expression of *hPARM-1* in *hPARM-1* non-expressing human cells.

Several studies showed matching results with our methylation-related genes. Paschalis et al. [28] addressed that the knockdown of *JMJD6* reduced prostate cancer cell growth, AR-V7 levels, and the recruitment of *U2AF65* to AR pre-mRNA. Mutagenesis studies suggested that the activity of *JMJD6* is pivotally associated with the generation of AR-V7 and with the catalytic machinery residing within a druggable pocket. Additionally, Dali Tong [29] proposed that targeting the *JMJD6*/*U2AF65* pathway may cause the inhibition of castration-resistant prostate cancer (CRPC) development.

As for the miRNAs, previous studies were used for comparison with our results, supporting the fact that the overlapping miRNAs we found were correlated with the regulation of PC. Kumar et al. [30] indicated that the reduction of *miR-1* and the elevation of *miR-21* were linked to biochemical recurrence in PC, suggesting that stromal miRNA expression may be informative for PC prognoses. Leite et al. [31] found that there was a significant global loss of *miR-143* expression during the transitions from high-grade prostate intraepithelial neoplasia to invasive adenocarcinoma and from localized to metastatic adenocarcinomas. Additionally, Szczyrba et al. [32] observed a tendency towards a lower expression of *miR-143* in both high-grade tumors and poorly differentiated tumors in PC. Gururajan et al. [33] demonstrated that *miR-379* plays an important role in PC biology by facilitating tumor growth, the epithelial-to-mesenchymal transition (EMT), and bone metastasis. More importantly, high-expressed *miR-379* was associated with the disease-free survival of PC patients. Taddei et al. [34] suggested that cancer-associated fibroblasts induce the downregulation of *miR-1247* in PC cells. Furthermore, *miR-1247* targets neuropilin (NRP)-1 and downregulates EGFR signaling, thus effecting the survival, invasion, and proliferation of cells.

Gao et al. [35] concluded that *miR-452-5p* is downregulated in PC, and might affect the progression of PC by interaction with target genes through several significant pathways. Kojima et al. [36] indicated that *miR-1* and *miR-133a* downregulations frequently occurred in PC, and that both function as tumor suppressors. When compared to non-PC tissues, expression levels of *miR-1* and *miR-133a* in PC were significantly lower.

In conclusion, we presumed that these overlapping features play crucial roles and are potential biomarkers in the recurrence incidence of PRAD cancer patients, since they rank as the top 100/50 selected features in both models, and the regulatory patterns were concordant.

### 4.2. Functional Pathway Analysis of the Top DEGs

**Figure 7:**
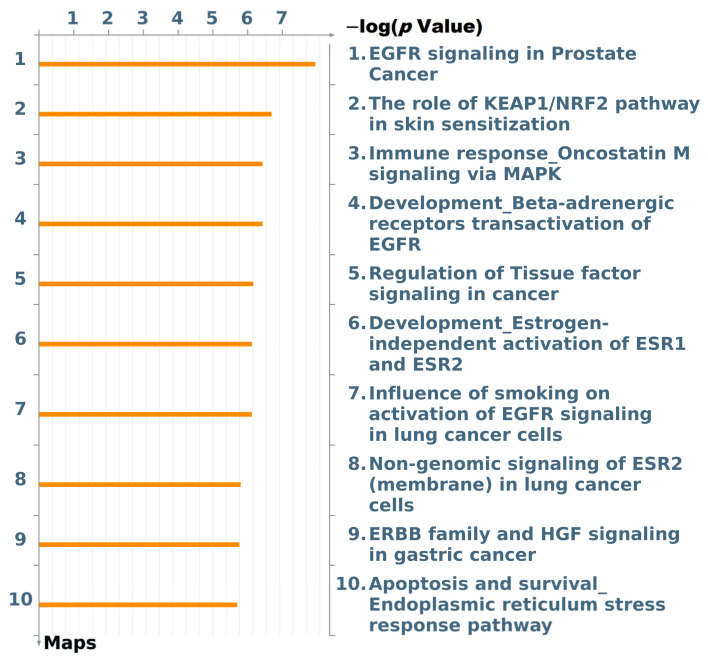
The map of top 10 pathways associated with the top differentially expressed genes. EGFR, epidermal growth factor receptor; KEAP1, Kelch-like ECH-associated protein 1; NRF2, nuclear factor erythroid 2-related factor 2; MAPK, mitogen-activated protein kinase; ESR1, estrogen receptor 1; ESR2, estrogen receptor 2; HGF, hepatocyte growth factor.

We used the MetaCoreTM-built network algorithm to discover which DEGs had direct interactions in our gene list (top 100 DEGs from the autoencoder model) and to conduct an enrichment analysis workflow to identify pathways in which these objects were involved. In Figure 7, among the top 10 pathways, the signaling process in epidermal growth factor (EGF) receptor (EGFR) was the most significant one associated with PC.

All EGFR family members are expressed in PC [37,38]. Within this family, ErbB2 is the favored dimerization partner for EGFR. The triggering of ErbB2/EGFR is connected to androgen-independent activation of the androgen receptor (AR) in PC [39]. Moreover, downstream signaling from EGFR may functionally inhibit the AR, even at the level of the plasma membrane. [40,41,42].

The overexpression of EGFR and the autocrine secretion of EGF and transforming growth factor (TGF)-alpha compose one of the key autoregulatory loops that facilitate the cell growth of a number of PC cell types. EGFR and its ligands, EGF and TGF-alpha, are overexpressed in PC during disease progression to more malignant hormone-independent and metastatic forms. The *EGFR* gene is amplified in many PC samples [43]. PC cells express the proto-oncogene *ErBB2*, as well as type III mutant EGFR, designated EGFRvIII, which mediates the cell growth of several human cancer cells [44,45]. ErbB2 is also often amplified in PC [46].

As shown in Figure S1, EGFR stimulation results in the activation of distinct intracellular signaling pathways, including the phosphatidylinositol 3-kinase (PI3K) cat class IA, extracellular signal-regulated kinase 1/2 (ERK1/2), phospholipase C (PLC)-gamma, and the signal transduction and activator of transcription 3 (STAT3) pathways [45]. Activation of the PI3K cat class IA/AKT (PKB) kinase and classical Ras/Raf/MEK/ERK1/2 cascades through EGF-EGFR leads to the downregulation of p27KIP1, which, in turn, changes the expressions of numerous mitogenic genes involved in PC cell growth. On the other hand, the inhibition of EGFR signaling cascades may cause the upregulation of the p27KIP1 protein, which then inhibits cyclin-dependent kinases followed by the G_1_/S transition of the mitotic cell cycle [47,48]. EGFR phosphorylates and activates STAT3, which, in turn, binds to and increases the transcriptional activation of the AR [49]. PLC-gamma is a downstream effector required for EGFR-mediated cell motility, which results in PC cell invasion and metastasis [50,51].

## 5. Conclusions

This is a study of recurrence-risk analysis and prognostic biomarker identification in PRAD patients after a radical prostatectomy. We implemented two computational analytical methods, the autoencoder model and similarity fusion network, to integrate three-omics data including gene expressions, methylation-related gene expressions, and miRNA. Both models differentiated significant subgroups of PRAD patients using multiomics features, but the autoencoder model generated better distinguishing power (C-index of 0.684). On top of that, our model could predict the recurrence rate once the related clinical data were obtained after the operation. In this regard, our proposed model not only possesses the compatible ability to distinguish low- and high-risk patients, but also the earlier prediction of the recurrence of PRAD, which would benefit patients by allowing for early interventions to prevent recurrence from occurring.

In addition, we built SVM classifiers with subgroups to predict the recurrence of PRAD. Finally, by testing the robustness through fivefold cross-validation, the SVM classifier using the autoencoder model-generated nodes outperformed other combinations with clinical information with 97.1% accuracy, 97.0% sensitivity, and 97.2% specificity.

Additionally, *TELO2*, *ZMYND19*, *miR-143*, *miR-378a*, cg00687383 (*MED4*), and cg02318866 (*JMJD6*; *METTL23*) were identified as highly associated with the recurrence-risk of PRAD patients. Multiomics panels were further constructed to evaluate the high- and low-risk subgroups. The outcome showed that the *p*-value = 5.33 × 10^−9^, C-index = 0.694, and C-index = 0.713, *p*-value = 2.97 × 10^−15^, respectively, for the multiomics panel and the other panel combined with the clinical information, which are better than the previous studies. This indicates that the omics features we selected are potential robust biomarkers for PRAD prognostic prediction. Additionally, we found pathways related to tumor development or prognosis via MetaCore, which indicates that EGFR signaling plays a vital role in the recurrence of PRAD.

This study contributes a different perspective to the current understanding of PRAD prognoses. In this regard, we suggest that these results should be taken into account for further clinical applications and better clinical decision-making.

## Figures and Tables

**Table 1:** Comparisons of studies integrating omics data for prognostic predictions.

Title	Cancer Type	Sample Size	Omics Data	Prediction Type	Methods	Reference
Predicting clinical outcomes from large-scale cancer genomic profiles with deep survival models	Pan-glioma (LGG/GBM), BRCA, KIPAN	Clinical and molecular data from TCGA	Gene expressions from TCGA	Survival analysis, established deep survival models to improve prognostic accuracy	Deep learning (DL) and Bayesian optimization methods	Yousefi et al., September 2017 [7]
Deep learning-based multi-omics integration robustly predicts survival in liver cancer	HCC	360 patients from TCGA	RNA sequencing, miRNA sequencing, methylation data (TCGA)	Survival prognostic predictions	DL-based model, validated on five external datasets	Chaudhary et al., March 2018 [8]
Deep learning-based multi-omics data integration reveals two prognostic subtypes in high-risk neuroblastoma	Neuroblastoma	407 patients from TARGET, 498 patients from SEQC	Gene expression, copy number alterations (TARGET and SEQC)	Identified two subtypes with significant survival differences	DL-based model, validated in two independent datasets	Li Zhang et al., October 2018 [9]
Integrative network analysis of TCGA data for ovarian cancer	Ovarian cancer	1214 Patients from TCGA	Gene expression, methylation data, miRNA, copy number alterations (TCGA)	Predicted clinical outcomes and elucidated interplay between different levels	A new graph-based framework	Zhang et al., December 2014 [10]
Similarity network fusion for aggregating data types on a genomic scale	GBM, BIC, KRCCC, LSCC, COAD	Patients ranging from 92 to 215 depended on cancer type profiled by TCGA	mRNA expression, DNA methylation, miRNA expression data (TCGA)	Prediction of patients’ survival risk analysis	Similarity network fusion	Wang et al., January 2014 [11]

TCGA, the Cancer Genome Atlas; miRNA, microRNA; TARGET, therapeutically applicable research to generate effective treatments; SEQC, sequencing quality control; LGG, low grade glioma; GBM, glioblastoma multiforme; BRCA, breast cancer; KIPAN, pan-kidney; HCC, hepatocellular carcinoma; BIC, breast invasive carcinoma; KRCCC, kidney renal clear cell carcinoma; LSCC, lung squamous cell carcinoma; COAD, colon adenocarcinoma; mRNA, messenger RNA.

**Table 2:** Performances of different hyper-parameter values of TCGA three-omics data.

	Epoch	Additional Hidden Layer Shape	Bottleneck Layer Shape	Normalization	Experiment	Survival-Related Node Number	3-Omics C-Index
No. of hyper-parameters used	5	500	200	Standard Normalization	DL	53	0.684 (SE 0.023)
No. of bottleneck nodes	10	500	100	Standard Normalization	DL	24	0.656 (SE 0.022)
	10	500	200	Standard Normalization	DL	59	0.668 (SE 0.023)
	10	500	300	Standard Normalization	DL	124	0.668 (SE 0.02)
	10	1000	100	Standard Normalization	DL	26	0.673 (SE 0.027)
	10	1000	300	Standard Normalization	DL	118	0.683 (SE 0.02)
	10	1000	500	Standard Normalization	DL	207	0.678 (SE 0.02)
No. of epochs	1	500	200	Standard Normalization	DL	70	0.677 (SE 0.02)
	5	500	200	Standard Normalization	DL	53	0.684 (SE 0.023)
	15	500	200	Standard Normalization	DL	83	0.676 (SE 0.026)
	30	500	200	Standard Normalization	DL	95	0.661 (SE 0.023)
	50	500	200	Standard Normalization	DL	124	0.663 (SE 0.027)
	1	1000	300	Standard Normalization	DL	115	0.679 (SE 0.02)
	5	1000	300	Standard Normalization	DL	103	0.672 (SE 0.02)
	15	1000	300	Standard Normalization	DL	120	0.666 (SE 0.023)
	30	1000	300	Standard Normalization	DL	162	0.659 (SE 0.023)
	50	1000	300	Standard Normalization	DL	188	0.67 (SE 0.023)
Hidden layers	5	1000, 500	100	Standard Normalization	DL	24	0.68 (SE 0.025)
	5	1000, 500	200	Standard Normalization	DL	86	0.668 (SE 0.023)
	5	1000, 600	200	Standard Normalization	DL	87	0.661 (SE 0.023)

DL, deep learning; SE, standard error.

**Table 3:** Fivefold cross-validation of the prediction accuracy of support vector machine (SVM) classifiers corresponding to different combinations of omics and clinical features.

Inputs for the SVM Classifier	Prediction Accuracy (%)	Sensitivity (%)	Specificity (%)
Autoencoder model-generated nodes (#53)	97.1%	97.0%	97.2%
Multiomics features (#250) ^1^	90.8%	93.8%	87.4%
Multiomics features + 3 clinical features ^2^	93.7%	95.6%	91.5%
Multiomics features + stage	94.3%	96.5%	91.7%
Multiomics features + age	90.7%	93.6%	87.4%
Multiomics features + Gleason Score	90.6%	93.2%	87.6%

^1^ Multiomics features included the top 100 differentially expressed genes, the top 100 differentially expressed methylation genes, and the top 50 differentially expressed miRNAs. ^2^ The three clinical features included were the Gleason score, age, and stage data.

## Data Availability

The datasets analyzed in this study are available from the Genomic Data Commons (GDC) Data Portal of National Cancer Institute (https://portal.gdc.cancer.gov (accessed on 15 November 2020)).

## Supplementary Materials

The following are available online at https://www.mdpi.com/article/10.3390/cancers13112528/s1, Figure S1: the signaling pathway of the epidermal growth factor receptor (EGFR). Table S1: demographics of patients included in the study, Table S2: top differentially expressed omics features derived from SNF, Table S3: top differentially expressed omics features derived from the autoencoder model. Table S4: the differential expression of the overlapped omics features between similarity network fusion and the autoencoder model.
