# Supplementary figures and images for: Biomarker Identification through Multiomics Data Analysis of Prostate Cancer Prognostication Using a Deep Learning Model and Similarity Network Fusion
Source: Cancers (Basel). 2021 May 21;13(11):2528. doi: 10.3390/cancers13112528 (PMC8196729; doi:10.3390/cancers13112528)

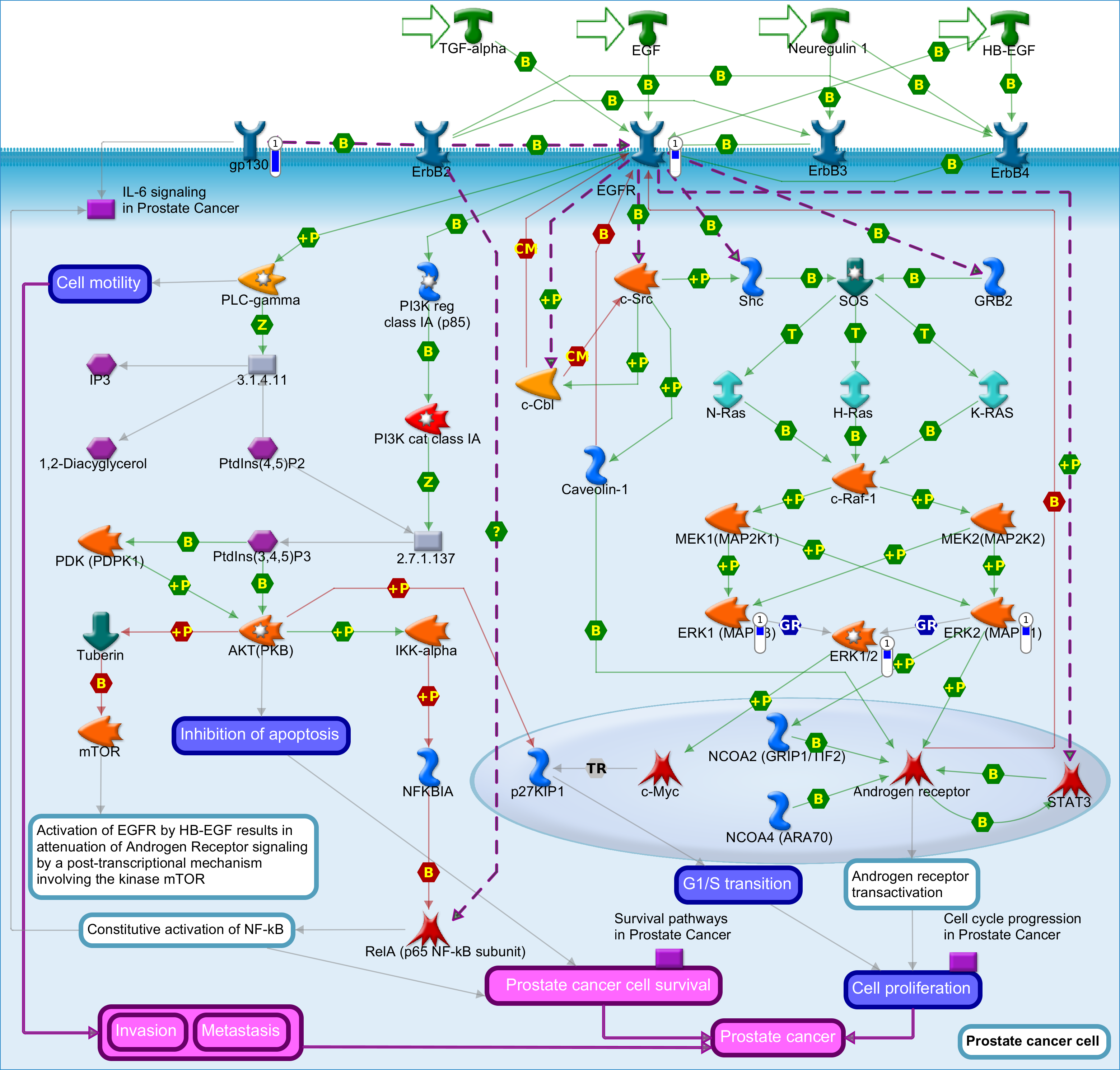

Supplement: Supplementary file 1 [file cancers-13-02528-s001.zip › FigureS1_The signaling pathway of the epidermal growth factor receptor (EGFR).png]
